# Consumer attitudes, barriers and facilitators to sharing clinical data for research purposes: Results from a focus group synthesis

**DOI:** 10.1016/j.heliyon.2024.e34431

**Published:** 2024-07-10

**Authors:** Richard J. Varhol, Crystal Man Ying Lee, Sharlene Hindmarsh, James H. Boyd, Suzanne Robinson, Sean Randall

**Affiliations:** a School of Population Health, Curtin University, Perth, Western Australia, Australia; b Silver Chain Group Limited (Silverchain), Perth, Western Australia, Australia; c School of Psychology and Public Health, La Trobe University, Melbourne, Victoria, Australia; d Deakin Health Economics, Deakin University, Melbourne, Victoria, Australia

**Keywords:** Consumer attitudes, Health data sharing, Health research, Lived experience, Data integration

## Abstract

Current research into the digital healthcare landscape reveals a significant gap in understanding the perspectives of consumers with lived health experiences on sharing their health data for research purposes. Despite the substantial value that such shared information can bring to healthcare research, policy development, and system improvement, insights into the attitudes and willingness of these consumers towards data sharing remain sparse. This study seeks to fill this gap, exploring the unique views of these individuals and assessing the potential benefits their data sharing could contribute to healthcare.

Utilising three focus groups, this qualitative study engaged 25 consumers with chronic health conditions to explore their attitudes towards sharing their personal health data for research. Conducted in Western Australia, the sessions were adapted to online and face-to-face settings due to COVID-19 restrictions. Discussions were recorded, transcribed and analysed using a reflexive thematic approach to capture the diverse perspectives and experiences of the participants.

The study revealed critical insights into how consumers with lived health experiences perceive the sharing of their health data for research. Key themes identified included data management and privacy concerns, the importance of transparent and patient-centric data governance models, and the varying levels of consumer trust in healthcare data-sharing processes.

This research provides valuable contributions towards developing a more inclusive and effective framework for health data sharing in research. By understanding the unique perspectives of consumers with lived health experiences, the study provides an understanding of enhancing consumer trust and participation in data-sharing initiatives. These insights are crucial for improving the quality and relevance of healthcare delivery, research, and policy-making, ultimately benefiting the broader healthcare sector.

## Highlights

• Investigated the perceptions of sharing health data for research purposes among 25 consumers with chronic conditions.
• Applied reflexive thematic analysis to analyse focus group discussions.
• Identified key themes such as data management and ethics, health system improvements and the desire for patient involvement.
• Offers insights into improving participation in sharing health data for research by promoting transparency and inclusivity.

## 1 Introduction

As healthcare systems embrace digital health technologies to address the needs of an aging population [1,2], they are confronted with an unprecedented volume of administrative and clinical data, accounting for an estimated 30 % of the world's total data volume [3]. The adoption of digital health solutions is evident in their rapid expansion across multiple sectors, including government agencies, public institutions, private enterprises, and individual consumers. This growth is driven by the promise of revolutionising healthcare delivery and management through reduced costs and increased accessibility, resulting in improved healthcare outcomes.

Although multiple platforms exist for collecting and storing data, few solutions are integrated or are capable of sharing data. Despite the World Health Organization advocating for the responsible sharing of secondary health data [4], this does not always occur in practice. Several factors contribute to fragmentation and limited sharing of health data. The lack of integration and interoperability among data platforms is one of the most significant barriers to realising the full potential of health data for public health monitoring, policy-making, and research.

In response to these challenges, there is a growing interest from both private and public sectors in harnessing the potential of these datasets through data linkage methodologies [[5], [6], [7]]. Data linkage, the process of combining data from different sources for the same individual or event to create a comprehensive dataset [8], offers a promising solution to overcome the barriers of data fragmentation [9,10]. By effectively linking disparate health data, researchers and policymakers can gain holistic insights, enabling more informed decisions and interventions in public health. The potential benefits of data linkage include enhanced accuracy in public health monitoring [11,12], more robust policy-making based on a broader range of data [13], and richer datasets for health research [14]. However, the success of data linkage relies on advanced technological infrastructure and rigorous standards to ensure data quality and consistency [15].

Public engagement is essential for building trust, addressing concerns, and ensuring that data sharing and linkage practices align with societal values. Public support is essential for secondary data use in health research, as evidenced by controversies over national data records systems in Denmark [16], England [17,18], certain European Union countries [19], Canada [20] and Australia [21], leading to a growing focus on ensuring that the public understands and supports these practices.

In Australia, as in many parts of the world, concerns related to public trust [[22], [23], [24]], informed consent [19,25,26], and minority inclusion arise when considering collecting and using health data for research purposes. Previous studies have demonstrated a positive correlation between public trust levels and the willingness to share health data for research [24,[27], [28], [29]]. However, the extent varies across geographic locations and demographics, requiring a context-specific approach for a deeper understanding of regional dynamics associated with the inherent complexities and fragmentation within the system.

Understanding the attitudes of consumers with a lived experience towards sharing clinical data is crucial and plays a pivotal role in achieving system efficiency and care integration. This highlights the need to address the impact of consumer perspectives on data sharing for secondary purposes, such as research and policy development. Addressing these concerns and gaining a deeper understanding of consumer attitudes has the potential to develop a more cohesive and effective healthcare system.

This study probes consumer perceptions and attitudes towards data sharing through focus groups to uncover the factors, challenges, and opportunities tied to integrating patients' administrative health and clinical data for research. It further contributes to a body of research into clinical perspectives [9] data governance [30] and consumer perceptions [31] of sharing data for research purposes. This qualitative inquiry seeks to understand the key elements influencing consumers' willingness to share their health data for research-related objectives.

## 2 Methods

Focus groups were used as the primary method to investigate the perceptions and attitudes of individuals with chronic health conditions towards sharing their personal health data for research purposes. The study was designed to gain a nuanced understanding of the factors influencing the willingness of these individuals to share personal health data for research purposes. This insight is valuable for shaping policies, interventions, and communication strategies that align with the preferences and concerns of this specific population. Recognised for its effectiveness in eliciting rich qualitative data, the Focus Group approach brought together participants with shared health experiences to engage in open dialogue within a structured yet adaptable setting [[32], [33], [34], [35]].

### 2.1 Interview guide

The research team performed an initial review of studies relating to consumer perceptions and attitudes towards the secondary use of medical data [[32], [33], [34], [35]]. This produced a semi-structured interview guide with open-ended questions relating consumer perceptions to sharing data for research purposes. The interview guide allowed for in-depth exploration of participants' attitudes, concerns, and perceptions related to sharing health data for research. It ensured that key topics were covered while providing the flexibility needed for participants to express themselves openly. Questions (Table 1) included topics associated with 1) who should have access to data for analytical purposes, 2) the type of information willing to be shared, 3) the purpose for sharing, 4) information governance, and 5) anticipated benefit. Survey questions were iteratively refined, with the Advisory Group consisting of consumer advocates and researchers.

**Table 1:** Group questions.

What are your thoughts about sharing your health data?
What health data would you be willing to share?
Who can use/access your health data?
Who can manage your health data?
What benefits would you anticipate from sharing your data?

### 2.2 Participant recruitment

An open invitation was sent to consumer advocate representatives based in Western Australia, who in turn posted the invitation to their relevant forums, inviting participants diagnosed or those with a lived experience with at least one of the three chronic conditions of interest (type 2 diabetes, chronic obstructive pulmonary disease (COPD) and heart failure) to attend a focus group session This resulted in a significant proportion of participants aged 55 and above, with 36.0 % being over 65 years old, enabling the perspectives of older adults with chronic conditions, a demographic with growing prevalence [36] and often underrepresented in qualitative health data research [37,38], to be adequately captured and incorporated into our study findings.

Participants interested in the study signed up through an online registration portal, after which a research team member contacted each registrant to explain the study's objectives, discuss the consent form, and schedule a suitable time for the focus group. Additionally, participants were asked a series of sociodemographic questions regarding gender, age, chronic conditions and their perceptions of managing their health. During these conversations, the study team utilised the snowball recruitment strategy [39], asking participants to recommend additional individuals they thought would be interested in joining the study. Participants who attended the events were provided a monetary compensation as per the Health Consumers' Council WA guidelines [40].

### 2.3 Focus groups

Focus group events were conducted between July 2021 and March 2022, and lasting approximately 90 min. Due to COVID-19 restrictions, two focus groups were hosted online using Microsoft Teams, with the last session being face-to-face. Registrants for the online sessions were provided with instructions on how to use the technology and asked before the meeting if they required assistance. All participants were provided with information about the project a week before the event.

The structure of each session involved an initial presentation delivered by RV on sharing data for the purposes of research, providing a patient case study and describing data linkage in the context of an integrated health system with associated examples of research applications. Following the presentation, participants were divided into break-out groups of three or four individuals. Each group was asked to answer four questions (Table 1) and encouraged to discuss their thoughts and perceptions on sharing health data for research purposes with each of the facilitators. For sessions with fewer than six participants, break-out groups were not formed and instead facilitated by RV, with SH and LT acting as either moderators or observers.

Discussions were digitally recorded with participants' verbal consent and supplemented by handwritten notes taken by the researchers (RV, SH & LT) to capture key points and nuances. After each session, the researchers synthesised the dominant themes, reflecting on any perceived personal biases, including the benefits and challenges of conducting the event online.

### 2.4 Data analysis

All event summaries were de-identified and transcribed verbatim by RV. NVivo [25] software was used for data organisation, management and analysis. A reflexive thematic approach was used as described by Braun and Clarke [24], allowing for the consideration of diverse viewpoints while recognising participants' unique backgrounds that influence their understanding and experiences [41]. The analysis process began with the creation of a provisional coding framework from a preliminary review of the transcriptions.

The research team collaboratively developed and continually refined the coding framework through iterative reviews and discussions, in line with the reflexive thematic analysis [42] ensuring it accurately represented the diverse viewpoints and complexities of the focus group data.

Both RV and SH independently assessed each transcript, identifying relevant quotes and creating descriptive codes. Subsequent team discussions helped reconcile and refine these findings, leading to a consensus on the initial coding framework. This collaborative analysis identified main themes and sub-themes, evolving from the constructs from Robinson's framework on trust, healthcare relationships, and chronic illness implications [23] and supported by illustrative quotes to contextualise the findings. While we acknowledge the possibility of overlooking subtle themes in our dataset of 25 participants, we are confident we have identified the major themes due to the consistency of patterns across the data.

### 2.5 Ethical issues and approval

All participants who attended the in-person event provided written informed consent on the day of the focus group, while those who attended the online sessions due to COVID-19 restrictions gave their consent verbally. Participants were informed that all their comments were confidential and that findings from the study may be published. Collected data were reported in such a way that persons could not be identified. Only researchers involved in the data analysis had access to the data. The study was approved by Curtin University's Human Ethics and Research Committee (Ref approval number: HRE2019-0619-011).

## 3 Results

**Fig. 1:**
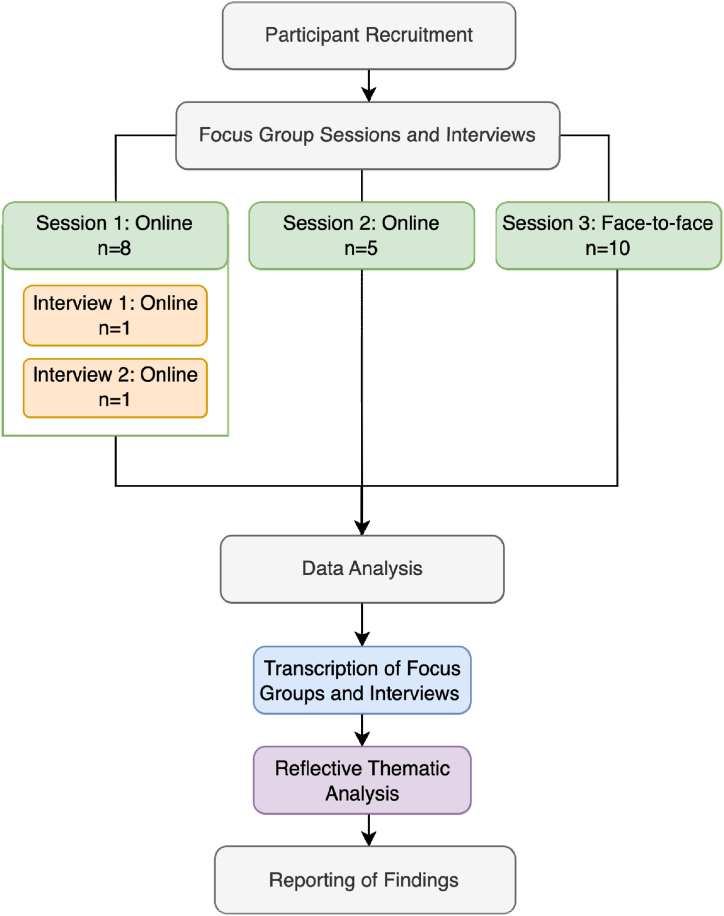
A flowchart depicting the research process of the study, from participant recruitment to data analysis and reporting.

A total of 23 participants took part in three focus group sessions, with attendance ranging between 5 and 10 in each (mean = 7.6). The initial focus group had 10 registrants; however, two participants did not feel comfortable attending on the day of the event and instead expressed an interest in being interviewed separately. Consequently, 8 participants fully engaged in the session. The two individuals who opted out were later interviewed individually for an hour using the same questions as in the focus group, though without the collaborative input and deliberation typical of the group setting. The second focus group saw the participation of 5 individuals. A third focus group was conducted to broaden the scope of perspectives, engaging 10 participants. Fig. 1 below illustrates the research process and the sequencing of activities.

Table 2 shows the majority of participants across all sessions were aged between 55 and 64 years (44 %; n = 11), with females having the most representation (76 %, n = 19). The majority of participants (72 %, n = 18) had obtained either a certificate-level education or a bachelor's degree, and just over half felt very confident in managing their own health (52 %, n = 13). 84 % (n = 21) of the participants indicated having at least one of the chronic conditions of interest. All participants resided within the metropolitan Perth region and had considerable lived experience of using the health system, with 4 indicating they were caring for someone with a medical condition.

**Table 2:** Participant demographics.

Variable	Session 1 n = 8	Session 2 n = 5	Session 3 n = 10	Interviews n = 2	Total N = 25
Age (years)					
45–54	2 (25.0 %)	2 (40.0 %)	0	1 (50.0 %)	5 (20.0 %)
55–64	5 (62.5 %)	3 (60.0 %)	2 (20.0 %)	1 (50.0 %)	11 (44.0 %)
65+	1 (12.5 %)	0	8 (80.0 %)	0	9 (36.0 %)
Gender					
Male	4 (50.0 %)	0	2 (20.0 %)	0	6 (24.0 %)
Female	4 (50.0 %)	5 (100 %)	8 (80.0 %)	2 (100 %)	19 (76.0 %)
Education					
Year 12	0	0	4 (40.0 %)	0	4 (16.0 %)
Certificate Level III or IV	2 (25.0 %)	1 (20.0 %)	4 (40.0 %)	2 (100 %)	9 (36.0 %)
Bachelor degree and above	6 (75.0 %)	2 (40.0 %)	1 (10.0 %)	0	9 (36.0 %)
Prefer Not to Answer	0	2 (40.0 %)	1 (10.0 %)	0	3 (12.0 %)
Medical Condition					
Yes	6 (75.0 %)	3 (60.0 %)	10 (100 %)	2 (100 %)	21 (84.0 %)
No	2 (25.0 %)	2 (40.0 %)		0	4 (16.0 %)
Confidence in managing own health					
Very confident	5 (62.5 %)	2 (40.0 %)	6 (60.0 %)	0	13 (52.0 %)
Somewhat confident	3 (37.5 %)	2 (40.0 %)	4 (40.0 %)	0	9 (36.0 %)
Moderately confident	0	1 (20.0 %)	0	2 (100 %)	3 (12.0 %)

Our analysis identified four overarching themes that captured healthcare consumers' attitudes toward the sharing and utilisation of their personal health data for research purposes (Table 3). These themes represent a spectrum of insights that reflect nuanced perceptions across the multifaceted dimensions of the healthcare data ecosystem, encompassing data management and ethics, health system improvement, inclusivity and information access, and stakeholder engagement and trust.

**Table 3:** Identified themes.

Main Themes	Associated Themes	Sub-themes
Data Management and Ethics	• Data Security and Privacy	• Concerns about Data Breach and Misuse • Autonomy in Data Sharing
	• Ethical Considerations and Consent	• Informed Consent and Ethical Standards • Ownership and Control of Data
Health System Improvement	• Data Utilisation and Benefits	• Direct Benefits to Patients • System Efficiency and Integrated Care
	• Community and Societal Impact	• Population Health and Research • Social Determinants of Health
Patient-Centric Approach	• Stakeholder Engagement and Trust	• Trust in Responsible Entities • Transparency and Accountability
	• Advocacy and Consumer Empowerment	• Role of Advocacy Groups • Empowering Patients as Data Consumers
Inclusivity and Information Access	• Accessibility and Health Literacy	• Navigating Health Information • Inclusivity and Support for Diverse Needs

The main theme of data management and ethics uncovered two associated themes of data security and privacy and ethical considerations and consent, which highlight the interplay of concerns and prerequisites that guide the willingness to share personal health information.

### 3.1 Data management and ethics

Participants relayed a complex landscape of concerns and expectations, articulating a heightened awareness and sensitivity towards data security, emphasising the potential for breaches and the misuse of their personal health information. The importance of personal data was a recurrent theme, with participants expressing a desire for robust security measures and stringent ethical standards to safeguard their information.

“The issue I have is having unknown people accessing my data and finding out who I am. I really don't want my [patient records] to go to the wrong person or have it accessed by the wrong people.” 1004-1

In their responses, participants highlighted concerns related to their personal health data, pointing out the risks of unauthorised access and misuse, necessitating the need for effective security measures and ethical guidelines to protect their sensitive information, reflecting a keen awareness of data protection issues.

“Say your research was on mental health … you would only expect that you would get mental health information, not cancer or any other data. It shouldn't be a broad overreach to get all the records. There should be something in there to limit and restrict what’s provided. And that it only covers your field of study.” 0314-3

Participants also suggest a preference for data-driven enhancements to healthcare quality and system efficiency by voicing support for a health information ecosystem that facilitates better care coordination and reduces systemic fragmentation. One participant expressed the value of data sharing in improving care delivery, highlighting a gap between current practices and the ideal state of integrated care.

“The best possible outcomes are from sharing our own data with our own healthcare team which doesn't currently happen." 0706-2-3

A sentiment further reinforced by the participant's perspective on the benefits of data sharing for personal health management suggesting that patient-centric data utilisation is pivotal for optimising health outcomes.

“If we are able to collect appropriate data and show that we delivered the care that patients were actually looking for, then we will be better equipped to understand whether the health system is delivering what is most valuable” 0706-2-3

Consumers also expressed an interest in having autonomy over their data as to who in their constellation of care has access and contributing to selected research initiatives.

“I think it would be awesome to access my data because I’d feel like I’m in control and can have the confidence or reassurance that I’ve put a block on that organization and only keep the appropriate people on my team that I want to have access to my data. If there's a research opportunity that comes up, I can unblock it and participate if I choose.” 1004-1

### 3.2 Health system improvement

Insights into the perceived impact of data sharing on patient care and overall system efficiency showed an expectation that data sharing should directly benefit individual patients and their desire for tangible outcomes from their contribution.

"I want to be benefiting as a patient. Not just be a footnote in some report … " 0924-2

Participants voiced frustrations with current inefficiencies and expressed sentiment for a more streamlined, patient-centred approach.

“I think it would be worth sharing information between different departments in hospitals and specialists, as long as the information is de-identified so that it cannot be traced back to individuals. This would help to improve the integration of the system, which is currently very dysfunctional.” 0706-2-2

The role of data sharing in enhancing population health and supporting research also resonated with participants recognising the potential for shared data to lead to healthcare innovations, with one individual expressing support for research, which was underpinned by an understanding that broad access to data could inform better healthcare strategies and outcomes.

“If this data linkage was going to go ahead, it would need to explain how helpful it could be and the cost savings. But certainly, the salient point is that it's going to help you navigate the health system and especially in the emergency department if all the data has been joined up and they've got all your records and your teams can talk together.” 0924-1

Social determinants of health emerged as a sub-theme, with participants acknowledging that healthcare extends beyond clinical interventions. The comprehensive care paradigm was described as essential for overall well-being, recognising the convergence of healthcare data with wider social factors.

“I would suggest that we need to decide how to explain to the general population that the health system is about looking after their well-being from the day they are born to the day they die. We need to explain that there are many benefits to pooling our data so that we can understand what is happening in the population, why it is happening, and what we can do to reduce co-morbidity, chronic illness, and so on. We can also use this data to improve social determinants of health.” 0706-2-1

### 3.3 Patient-centric approach

In exploring the patient-centric approach to healthcare data management, participants identified a pressing need for a healthcare system that not only respects but actively involves patients in the management and utilisation of their health data. Themes related to the dynamics of stakeholder engagement and trust, alongside advocacy and consumer empowerment, were observed. The theme of trust in responsible entities was noted by participants, who underlined the importance of transparent and accountable data practices illustrating the necessity for patient-centred care approaches.

"I'm a huge advocate for the health system actually delivering on what patients need, rather than what the system has decided is right for them." 0706-2-3

Trust in responsible entities also emerged, with one participant identifying the necessity for transparent and accountable data-handling processes and the importance of clear communication in fostering confidence in data management systems.

“Transparency, honesty and just getting a better understanding of where the data is going and how it fits in with the [health] system itself is really important. I have absolutely no problem with data sharing provided the system can explain to me clearly why and how they're going to use my data and that my privacy is protected.” 0706-2-3

Participants voiced a desire for active participation in decisions regarding their health data, highlighting the need for patient agency in healthcare processes.

“It's more than just a [prescription]. It's more than just a doctor. It's someone to help you exercise. It’s someone to talk to. We need someone to help us look through all our activities and the associated finances with the family - that sort of thing. That's what makes the whole person. It’s looking at the whole picture, not just focusing on a doctor for help.” 1004-1

The role and involvement of advocacy groups were seen as a means to enhance the transparency and accountability of the organisations involved in the data-sharing process. Participants noted that these groups can represent patient interests, ensuring that data management practices align with the needs and preferences of healthcare consumers at the same time expressing a desire for greater involvement in decisions affecting their data, and the importance of patient voices in developing a transparent and integrated healthcare system.

“Advocacy groups should be made up of consumers, carers, support groups and the like … I think you'll find more gets done when it comes from your consumer because we have a vested interest in outcomes, of course.” 0924-2

### 3.4 Inclusivity and information access

Health literacy and access to health information was an underlying theme across all participants who highlighted a digital literacy gap, especially among older populations, simultaneously identifying the need for health information to be accessible and understandable to all. Participants pointed out the challenge faced by the elderly in adapting to digital health platforms and the necessity for support mechanisms tailored to diverse needs in navigating health data.

“To do all this stuff and have digital records you need to have skills in using computers and databases and stuff like that. A lot of older folk just aren't computer literate. It's got to be all-inclusive.” 0924-1

“A lot of people my age or older, do. They are just not coming to terms with the technology. A lot of them just want to be left alone with the way they’ve always done things” 0706-1.

These statements underscore the resistance encountered among certain groups towards technological advancements in healthcare, which can however be influenced by a diagnosis.

“Look, when people have been diagnosed with chronic conditions, I think is very different. It’s like you become an expert in your chronic conditions. Your health literacy ends up being incredibly high because you're living and breathing your chronic illness all the time.” 0924-3

Participants revealed a multifaceted perspective on healthcare data management, highlighting the need for stringent data security, ethical practices, and calls for improved health system efficiency and patient benefits. Consumers and carers advocated a patient-centric approach, underscoring the importance of stakeholder trust, transparency, and patient empowerment. Additionally, the necessity for inclusive information access and enhanced health literacy emerged as a crucial aspect for ensuring equitable healthcare experiences for all individuals.

## 4 Discussion

Our findings uncover a multifaceted and complex ecosystem providing insights into health consumers' perspectives on sharing and utilising their personal health data for research. These are characterised by a dynamic interplay among data management, system improvement, patient-centric approaches, and inclusivity.

Central to these findings is the theme of data management and ethics, revealing an uneasiness associated with data breaches and misuse and echoing the findings of similar studies [29,43]. This apprehension underscores the necessity for building trust through transparent and robust data security and privacy measures, aligning with the growing literature advocating for informed consent and ethical standards in health data management [[31], [32], [33],^35^,^44^]. Such concerns are magnified by the limited understanding amongst consumers about how their health data is utilised and managed, highlighting a gap between advocacy for responsible data sharing and the actual comprehension of these practices by consumers. The need for refined, patient-centred data governance models [[45], [46], [47]] is underscored by the importance of maintaining autonomy in data sharing and controlling personal health information, a process that is influenced by consumer awareness and understanding of the potential individual and collective benefits of health data use and management [19].

From a health system improvement perspective, participants recognised the potential benefits of data sharing in terms of enhancing patient care and system efficiencies. However, they expressed a preference for data to be used for direct benefits for their individual health. This priority contrasts with their limited interest in data usage at a population level. This position suggests that despite previous studies highlighting the potential of data sharing to optimise healthcare delivery and reduce systemic fragmentation [[48], [49], [50], [51]], data integration for research purposes [31,52,53] is not receiving the attention it should.

The importance of a patient-centric approach, suggested stakeholder engagement and trust, as being crucial for encouraging consumers to share their health data for research. Participants’ desire for transparency and accountability in data handling processes aligns with existing literature on patient trust and its impact on data sharing willingness [26,54]. Earlier studies found a majority (80 %) of Australians prefer healthcare providers over the Commonwealth government for managing personal information for research [55,56]. Our findings, however, further delineate management from analysis, with consumers favouring the government entities to manage their data while preferring healthcare providers for data analysis [57], suggesting a shift in trust to the larger government departments associated with the rise in cyber incidents. Concurrently, advocacy and consumer empowerment; were found to be a recurring pivotal theme in integrating patient perspectives into healthcare decision-making processes [58], highlighting an area for broader exploration.

Most (52 %) of the study's participants reported high confidence in managing their health, suggesting both a higher level of health literacy as well as a broader engagement with the health system and a greater willingness to share their data. This underscores the importance of inclusivity and information access in healthcare, with a growing need for accessible and user-friendly health data systems to accommodate diverse patient needs, especially among older populations or those with limited health literacy. Low health literacy, often linked to limited understanding of health issues and challenges in navigating healthcare systems, poses a significant barrier to enhancing care quality and health outcomes, particularly in disadvantaged groups [[59], [60], [61], [62]]. This finding is consistent with previous research pertaining to the importance of designing health systems that are user-friendly and accessible to all, regardless of their technological proficiency or health literacy levels [63].

Our study also identified a divergence in the willingness to share health information based on educational attainment. Previous studies showed that participants with less than a bachelor's degree are more reluctant to share health information and medical records than those with higher educational qualifications [64]. Even though our study had a smaller cohort of participants, most of whom had less than a bachelor's degree (52 %), this contradiction suggests varying levels of trust or understanding of data use across the healthcare continuum, indicating a complex relationship between health literacy, understanding how their data is used, and trust in health data sharing initiatives.

The findings highlight the importance of engaging with consumers and enhancing their awareness of the advantages of data sharing for research purposes. Working with consumers on the utilisation of their data and ensuring their understanding of the benefits, especially from a population health perspective, is pivotal in bridging gaps in consumer knowledge and aligning data-sharing practices with their expectations. Such initiatives can lead to a more informed and involved public, which is essential for the effective integration of patient data into healthcare delivery and research and plays a critical role in enhancing consumer trust and participation in data-sharing initiatives.

## 5 Limitations

Although our study, provides useful insights from a vulnerable Australian context, several limitations require consideration. Firstly, the advent of COVID-19 significantly disrupted the planned structure of the focus groups, requiring isolation protocols to be followed. This necessitated the study to pivot from in-person to virtual sessions, resulting in fewer participants which may have influenced the depth and nature of the discussions. Additionally, these discussions may have been affected by the various data breaches [65,66] that occurred over the time period between focus group sessions. Secondly, a selection bias may have inadvertently been introduced through the participant selection process which recruited individuals with specific lived experiences as part of a broader study [57]. People who responded to the invitation to participate were generally older, this reflected the increased prevalence of the chronic conditions of interest in the aging population [36]. Therefore, we are unsure if younger adults with chronic conditions have similar views towards sharing clinical data for research. Furthermore, participants were compensated for their involvement, thereby attracting candidates who were potentially more engaged in research activities resulting in a sample size and demographic composition not representative of the broader population. Finally, the use of focus groups, while valuable for qualitative depth, limits the breadth of the captured perspectives as well as introduces subjectivity which may have impacted the neutrality of the data.

## 6 Conclusions

This research, situated within the Australian healthcare context, delineates a complex interplay between public trust, autonomy, and the inclusivity of consumers in health data sharing for research. Our findings reveal that consumers exhibit a conditional willingness to engage in data-sharing activities predicated on solid data protection practices and a clearly defined purpose for the use of their data. While the potential for enhanced individual and system-wide health outcomes is recognised, there is a pronounced call for greater control and transparency. The insistence on a clearly defined purpose for data use reflects this desire for transparency, enabling consumers to make informed decisions about participating in data-sharing activities. Informed decision-making is a key theme, highlighting consumers' desire to understand how their data will be used and the influence of presented conditions on their choices.

The increasing emphasis on data protection practices among consumers is indicative of a growing awareness and concern for safeguarding personal information, echoing a broader trend of heightened public sensitivity to data breaches and privacy issues in the digital era. Trust plays a pivotal role in consumers' willingness to share data, emphasising the need for organisations to prioritise secure and responsible data handling.

Additionally, the study highlights the importance of robust data governance, stakeholder analysis that acknowledges the lived experiences of healthcare consumers, and transparent communication detailing the intended benefits and uses of data linkage. This research also underscores the significance of health literacy and a need to improve consumer understanding of how their data is used, advocating for approaches that accommodate the diverse understanding and experiences of consumers. This includes involving consumers in governance roles and other key research and awareness-raising roles regarding health data management thereby aligning data-sharing initiatives with the needs of the public.

These insights are particularly relevant in the context of fostering an environment where trust, autonomy, and inclusivity are paramount in encouraging the participation of individuals in data-sharing for research, ensuring that the strategies developed are not only technically and ethically sound but also connected to the personal narratives of those whose data is being shared.

## Funding statement

This project was supported by the 10.13039/501100021841Western Australian Health Translation Network and the Australian Government's Medical Research Future Fund (MRFF) as part of the Rapid Applied Research Translation program.

## Ethics statement

Informed consent was obtained from all participants. Participants were informed that all their comments were confidential. Collected data were reported in such a way that persons could not be identified. Only researchers involved in the data analysis had access to the data. The study was approved by Curtin University's Human Ethics and Research Committee (Ref approval number: HRE2019-0619-011)

## Data availability statement

The data that has been used is confidential.

## CRediT authorship contribution statement

**Richard J. Varhol:** Writing – review & editing, Writing – original draft, Validation, Resources, Project administration, Methodology, Investigation, Funding acquisition, Formal analysis, Data curation, Conceptualization. **Crystal Man Ying Lee:** Writing – review & editing, Supervision, Investigation, Data curation. **Sharlene Hindmarsh:** Writing – review & editing, Validation, Investigation, Formal analysis, Data curation. **James H. Boyd:** Writing – review & editing, Supervision, Methodology, Funding acquisition. **Suzanne Robinson:** Writing – review & editing, Supervision, Methodology, Funding acquisition, Conceptualization. **Sean Randall:** Writing – review & editing, Supervision, Methodology, Investigation.

## Declaration of competing interest

The authors declare that they have no known competing financial interests or personal relationships that could have appeared to influence the work reported in this paper.
